# Laboratory and Field Evaluation of Graphene Oxide and Silver Nanoparticle-Enhanced Silicone Fouling Release and Biocidal Coatings for Marine Antifouling

**DOI:** 10.1021/acsomega.5c09101

**Published:** 2026-01-21

**Authors:** Michael R. Kelly, Olaug M. Aalen, Ingrid G. Hallsteinsen, Hilde L. Lein

**Affiliations:** Department of Materials Science and Engineering, 8018 Norwegian University of Science and Technology NTNU, Sem Sælands vei 12, 7034 Trondheim, Norway

## Abstract

This study explores the enhancement of silicone-based fouling release coatings through the incorporation of graphene oxide and silver nanoparticles. Laboratory tests demonstrated significantly improved microfouling resistance with increasing nanoparticle concentrations while maintaining surface energies within the optimal range for fouling release. During a 7 month marine field immersion trial, only the silver nanoparticle-containing composite coating exhibited improved antifouling efficacy over time, while the performance of the graphene oxide-containing composite coating was comparable to the simplified nonbiocidal fouling release coating. Both composite coatings showed a slightly earlier onset of macrofouling. Microscopy revealed notable nanoparticle agglomeration and localized algal attachment, emphasizing the need for improved dispersion and surface integration. Though the addition of nanoparticles for boosting antifouling efficacy with both biocidal and fouling release strategies showed only marginal improvements, clear evidence of enhanced performance is seen. Advancing this technology demands targeted control over nanoparticle distribution and leaching, which are key challenges that must be addressed to unlock the full potential of next-generation, sustainable antifouling coatings.

## Graphical abstract

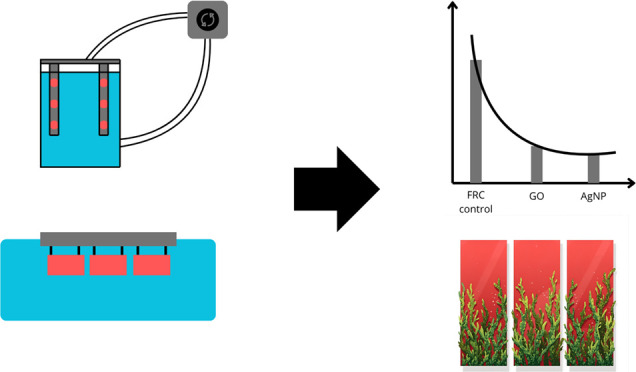

## Introduction

Biofouling can negatively affect the operational capacity of marine vessels by increasing the weight of the vessels and the surface roughness of the hulls, leading to increased drag, decreased speed, and higher fuel consumption.^1,2^ Biofilms form on these surfaces in a stepwise process. Initially, free-floating microorganisms such as bacteria attach to the surface using weak interactions. This stage is reversible and influenced by the surface properties and environmental conditions. Microorganisms produce extracellular polymeric substances that enhance their adhesion to the surface, making the attachment irreversible. This marks the beginning of the biofilm formation. The attached microorganisms proliferate, forming microcolonies that communicate and grow into mature biofilms with channels for nutrient and waste flow. The extracellular polymeric substance matrix protects the biofilm, and some cells detach to colonize new surfaces, continuing the biofilm cycle.^3^ Efforts to reduce biofilms target the initial stages to prevent algae adsorption.

Since 2000, there have been two different technologies dominating the fouling protection coating market, self-polishing coatings (SPCs) and the fouling release coatings (FRCs). The SPC technology is based on the controlled release of biocides, like copper with/without cobiocides, using a mixture of acrylic and natural binders as the delivery system. The FRC technology is based on PDMS, which gives a low surface energy that is difficult for fouling organisms to attach to. During the past decade, a third technology has emerged, the biocidal FRC, which is a hybrid of the SPC and FRC technologies. These materials feature low surface free energy, which helps reduce biofouling.^4,5^ Research has shown a direct relationship between the relative adhesion strength of foulants and surface energy.^6,7^ An optimal range for surface energy to reduce adhesion strength is between 20 and 30 mJ/m^2^. Low surface energy materials, such as silicones and fluorocarbons, are commonly used in the manufacturing of FRCs.^7^

Even though FRCs work well on some ships/trades, they tend to perform poorly in static water or at low speeds.^8,9^ Furthermore, they tend to have poor mechanical properties^10^ and thus short lifespans in harsh environments. Nanoparticles can be added to increase the mechanical strength of polymer coatings^11,12^ and add bactericidal properties to the composite coatings, which may improve the antibiofilm properties.^13^ Silver nanoparticles (AgNPs) have great cytotoxicity against a broad spectrum of microorganisms.^14^ The Ag^+^ released by the AgNPs can interact with the bacterial membrane and penetrate the cell by destabilizing it, followed by denaturation of proteins, damaging the DNA, and inhibiting bacterial propagation.^15–17^ Graphene nanomaterials have also been shown to be efficient in preventing the formation of biofilms.^18–20^ The mechanisms behind these are not yet fully understood due to their complexity and the wide array of factors that might affect their antibacterial activity,^21,22^ though popular theories include sharp edges of graphene sheets that puncture or otherwise damage the cell membrane,^23^ cell entrapment,^24^ and the formation of reactive oxygen species.^25^ These nanoparticles have potential as contact-killing biocides in polymer–nanoparticle composite coatings. AgNPs and GO in marine antifouling coatings present nuanced environmental risks, with emerging evidence suggesting limited acute toxicity but potential for chronic sublethal effects.^26,27^

Combining graphene nanomaterials with silicone polymers has proven to not only improve the mechanical strength but also improve the antifouling efficacy of composite membranes in both static and dynamic conditions.^28,29^ The effect of adding biocidal nanoparticles to FRCs could increase the static antifouling efficacy and the mechanical durability of the composite coatings. There is also increased focus on modified or functionalized graphene materials,^30–34^ though the antibiofilm performance of graphene oxide (GO) polymer composites is still unclear.^35^ Furthermore, antifouling studies have primarily been done for short immersion durations and in hydrodynamic conditions that do not necessarily mimic real conditions.^36^ The growth of filamentous algae is very common in the fjords of Norway. Green filamentous algae typically thrive in late spring and early summer, while brown and red filamentous algae tend to grow during the summer months.^37^ If biocide exposure is low and an initial biofilm and microalgae are present, these species can resettle on the surfaces due to their rapid regrowth abilities.^37^ GO and AgNPs have demonstrated effectiveness against early settlement and microalgae through biocidal contact killing. However, there has been limited research on their effects specifically related to preventing macroalgal growth.

In this study, we aim to compare the controlled laboratory experiments with real-life applications by testing the same coatings with complementary biocidal and fouling release properties in both lab and natural marine conditions and investigate some of the factors that affect the antifouling performance. The performance against microfouling is tested using a *Phaeodactylum* sp. dominated algae culture in a bioreactor in controlled laboratory conditions. The performance against macrofouling is tested during long-term static field immersion in real conditions. The changes in the surface energies are investigated through drop shape analysis, and the particle dispersions in the nanocomposite coatings are investigated through confocal laser scanning microscopy.

## Materials and Methods

### Materials and Synthesis

The simplified nonbiocidal FRC, which is a commercially available FRC, was provided by Jotun AS (Sandefjord, Norway). GO paste (10 wt % in H_2_O) was acquired from CealTech AS (Stavanger, Norway). AgNPs (particle size <100 nm) were purchased from Sigma-Aldrich (Saint-Louis, USA). Xylene (100%) was purchased from Merck Life Sciences NV (Amsterdam, Netherlands). Polyethylene (PEHD) substrates (12.6 mm diameter, 4 mm thickness) were provided by the NTNU Workshop. A set of PVC panels (30 cm × 20 cm × 0.3 cm) pretreated with a primer coat and an intermediate tie coat were also provided by them, along with a bar applicator with a width of 20 cm and height of 400 μm.

For the FRC control samples (the simplified nonbiocidal FRCs), the provided components were mixed as per the product instructions before application with a bar applicator. For the FRC-nanocomposites, GO paste and AgNPs were added to the simplified nonbiocidal FRC to a final concentration of 0.500 wt % in the polymer and mechanically stirred before deposition on the pretreated PVC panels. For laboratory tests, GO and AgNPs were mixed to final concentrations of 0.125, 0.250, and 0.500 wt % in the polymer and mechanically stirred, along with xylene (70 wt % solvent), before spray deposition on PEHD substrates. Spray deposition was the preferred deposition method for the PEHD substrates due to their geometry and small size.

The spray deposition of the slurries was done using an Airbrush paint gun with a 0.3 mm nozzle with a nitrogen gas pressure of 2.0 bar. Before the deposition, the substrates were sonicated in ethanol for 5 min to clean the surface and then subsequently dried in a fume hood. The slurries were sonicated for 5 min to redisperse any precipitated solutions. The substrates were placed on aluminum foil during the deposition process. The paint gun was held at approximately 10 cm distance from the sample during the deposition. The samples were left in the fume hood overnight for the solvent to evaporate, and then, the process was repeated for a total of three applied layers for a final dry film thickness of approximately 400 μm.

The bar applicator was used for depositing on the panel samples for field testing. The applicator was put on the end of the plate, and the coating slurry was deposited in the applicator, which was subsequently pulled at a steady and even pace across the surface of the panel for about 10 s, leaving a wet film thickness of 400 μm. The coating was left to cure at room temperature overnight. The back sides of all of the panels were coated with a commercially available marine paint from Jotun AS by coating specialists at Jotun AS.

The mixed diatom cell culture was provided by an NTNU SeaLab. Conwy nutritional medium and silicate nutritional solution (Na_2_SiO_3_·5H_2_O) were also provided by NTNU SeaLab. Algae suspensions were grown in autoclaved and filtered seawater with additions of the growth medium.

### Antifouling Efficacy

Antifouling efficacy of the samples was conducted using a bioreactor, as shown in Figure 1. The bioreactor was prepared by the NTNU Workshop.

**Figure 1:**
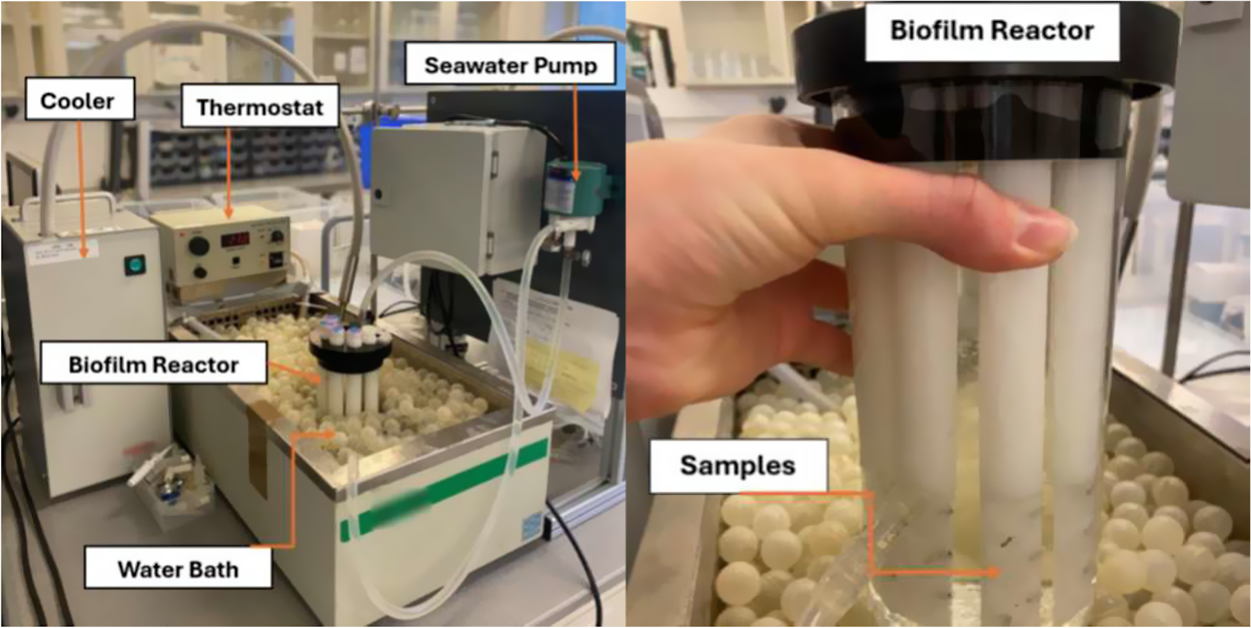
Experimental setup for the biofilm reactor experiment for antifouling efficacy testing. The image includes the bioreactor along with the water bath, cooling, and heating system, as well as the seawater pump.

700 mL of seawater containing the *Phaeodactylum*-dominated algae culture was run through the bioreactor, with samples mounted on the rods submerged in the bioreactor. The seawater pump was connected to the bioreactor through plastic tubes with a pump speed of 1 L/min. The experiment was conducted over 3 weeks in the mixed algae culture. After the samples were removed and gently rinsed in deionized water to remove salt particles, the samples were left to dry in a fume hood overnight before imaging. Triplets of each sample were tested. The antifouling efficacy of the samples was quantified by manual counting of cells adhered to the surface with the use of optical imaging using an Alicona Infinite Focus SL optical microscope with a 50× magnification lens. A set of ten images was taken on each sample, and the diatom growth was expressed as the density of diatoms on a total area of 0.166 mm^2^.

Field testing was done over a period of 7 months from November to June on floating rafts in the harbor of Sandefjord, Norway. The panels remained consistently submerged in the seawater 30 cm below the surface during the observation period and only raised from the water when imaging the sample surfaces. The panels were photographed after 28, 62, 91, 98, 162, and 209 days of immersion, roughly once a month, with the exception of March and May due to scheduled annual leaves.

### Material Characterization

The nanoparticle dispersion in the nanocomposite coatings was investigated by using a Zeiss 700 confocal laser scanning microscope. A 13 × 13 tile scan was performed using a 40× water immersion objective. A 639 nm laser source and a DAPI filter were used to image the nanoparticles. A 405 nm laser source was used to excite chlorophyll A for cell imaging with a BP filter of 650–700 nm.

Contact angle measurements were made using the sessile drop technique with a Krüss DSA100 Drop shape analyzer, with Krüss ADVANCE software for measuring the contact angles. Water was used as the liquid. The contact angles were averaged over 3–5 parallel measurements at different positions on the surface. The Young–Laplace method was used as the fitting method for the measurements. Diiodomethane was also used in order to obtain the contact angle measurements of a dispersive liquid, as well. Contact angle measurements were subsequently converted to surface free energy using the OWRK method,^38,39^ using eq 1.

$$\sqrt{\gamma_{\mathrm{sv}}^{d}\gamma_{\mathrm{lv}}^{d}}+\sqrt{\gamma_{\mathrm{sv}}^{p}\gamma_{\mathrm{lv}}^{p}}=0.5\gamma_{\mathrm{lv}}(1+\cos\theta )$$ 1

Here, the θ is the angle between the solid and the liquid, γ_lv_ is the total surface energy, and γ_sv_^d^, γ_lv_^d^, γ_sv_^p^, and γ_lv_^p^ are the dispersive and the polar parts of the solid–vapor and liquid–vapor surface tension, respectively. The surface tension components of the liquids are given in Table 1. Water was chosen as the polar liquid, and diiodomethane was chosen as the dispersive liquid.

**Table 1:** Surface Tension Components of Water and Diiodomethane

liquid	dispersive, γ_lv_^d^ [mN/m]	polar, γ_lv_^p^ [mN/m]	total, γ_lv_ [mN/m]
diiodomethane	50.8	0	50.8
water	19.9	52.2	72.1

## Results

### Surface and Particle Characterization

The surface energies of the coatings were determined through contact angle measurements using the OWRK method, and the results are shown in Figure 2. Detailed contact angle values can be found in the Supporting Information 2.

**Figure 2:**
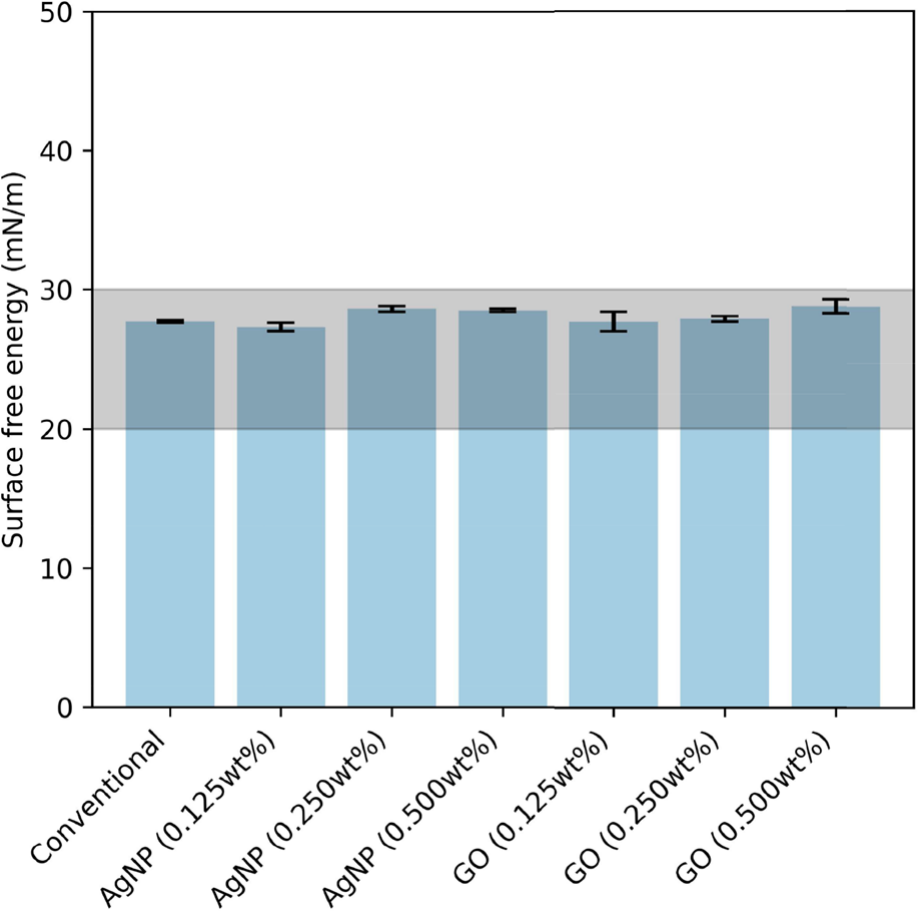
Surface energy of the simplified nonbiocidal FRC (Conventional) and the FRC composites with 0.125, 0.250, and 0.500 wt % AgNP and GO. Data represent the mean ± standard deviation from three independent replicates. Data represent mean ± standard deviation from three independent replicates. The grayed-out area of interest is the region associated with fouling release.

There is a slightly increasing trend in the surface energies for increased nanoparticle concentration, for both AgNPs and GO. However, the surface energies are still approximately around 27–28 mN/m. The mean of the samples is at 28.2 mN/m, with a very low variability (standard deviation of 0.575 mN/m, coefficient of variation of 2.0%). These values fall within the range of 20–30 mN/m, which is generally associated with effective fouling release performance.^4,40^ In this range, bacterial adhesion is minimized, allowing deposits to be easily removed by hydrodynamic shear. The addition of the nanoparticles to the coatings did not affect the coatings’ surface roughness.

The roughness of the coatings was assessed using an optical microscope (Table S3) and further examined with scanning electron microscopy (Figure S1). While the surfaces displayed some roughness, the addition of nanoparticles did not significantly influence the coating roughness.

The nanoparticle dispersion in the top layer of the coating is depicted in Figure 3. The surfaces with 0.125 and 0.500 wt % AgNPs are shown in Figure 3a,c, respectively, while the surfaces with 0.125 and 0.500 wt % GO are illustrated in Figure 3b,d. In the images, the red signals represent the nanoparticles, while the green signals indicate algal cells. The 0.125 wt % AgNP sample displays a dispersion of particles, with sizes ranging from a few micrometers in diameter to larger clusters of nearly 100 μm. The 0.500 wt % AgNP sample exhibits significant agglomeration, with particle sizes generally exceeding 100 μm. The 0.125 wt % GO sample demonstrates levels of agglomeration that look sheet-like, with particle sizes varying from just a few micrometers to several hundred micrometers. The 0.500 wt % GO sample contains some larger agglomerates as well, but it also has a considerable number of smaller particles.

**Figure 3:**
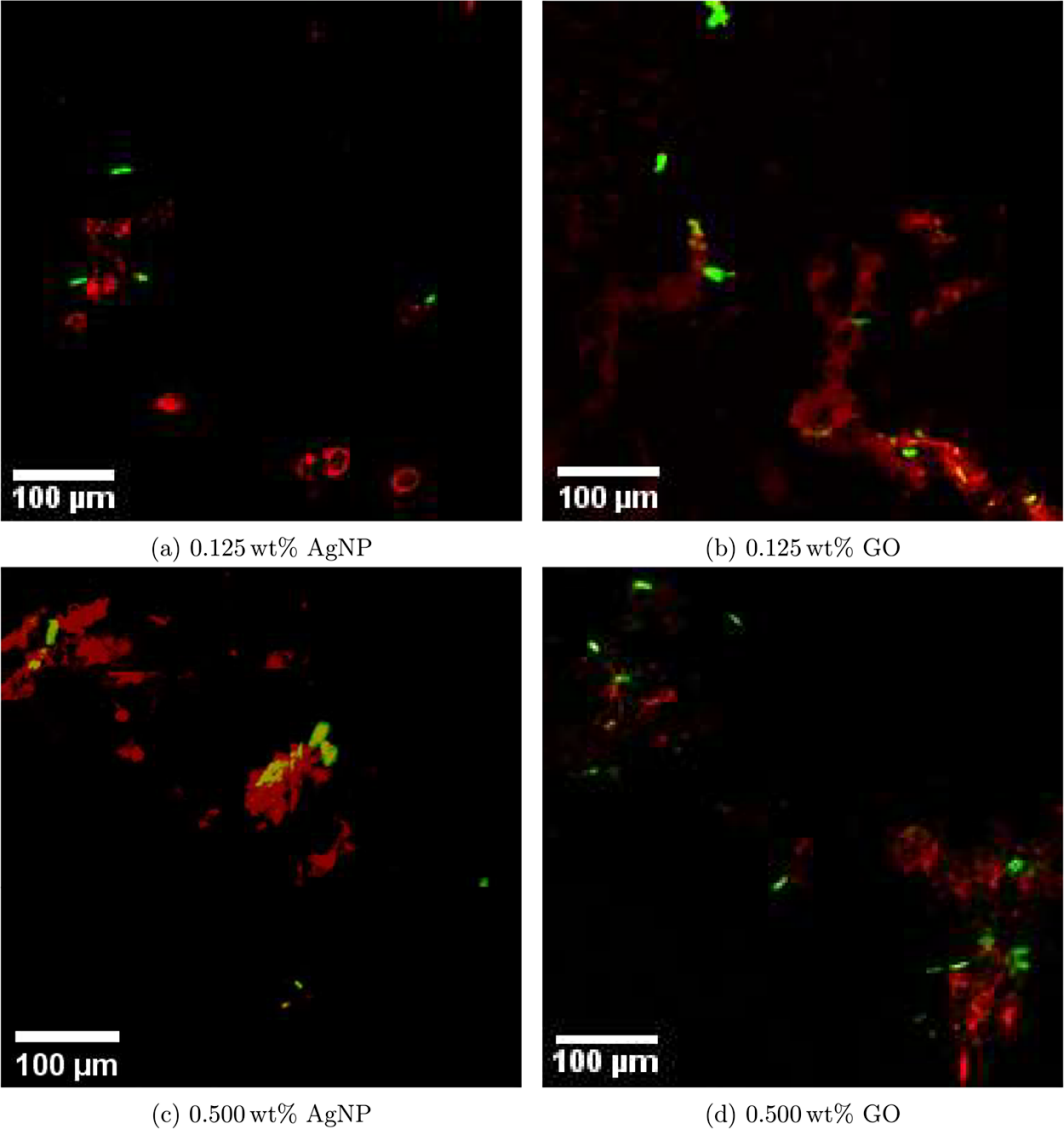
Confocal laser scanning microscopy images showing the distribution of nanoparticles (red) and diatoms (green) on the surface of the nanocomposite FRC after bioreactor exposure. (a, c) AgNP coatings at 0.125 and 0.500 wt %, respectively. (b, d) GO coatings at 0.125 and 0.500 wt %, respectively.

The algal cells exhibit the characteristic oval shapes typical of the *Phaeodactylum* sp.-dominated marine diatom culture used in the exposure tests, with a typical cell length of 5 to 10 μm. There is a trend of algal cells attaching to the surfaces near or on the nanoparticles for all samples.

### Antialgal Performance

The algal cell density on the sample surfaces after 3 weeks of immersion in a bioreactor is shown in Figure 4. The uncoated PEHD substrates exhibited the highest level of fouling, with a mean algal density of approximately 60 mm^–2^. The significant variability in algal cell density on the uncoated PEHD surface can be attributed to the heterogeneity of fouling settlement and the presence of cell clusters.

**Figure 4:**
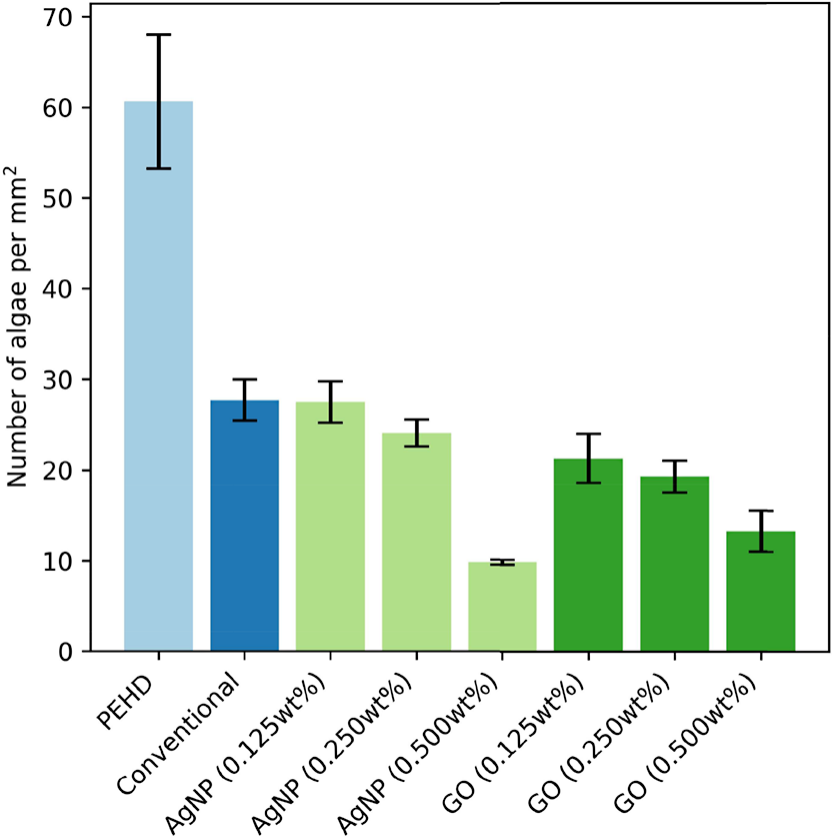
Mean cell density of algae on sample surfaces after 3 weeks of immersion in the bioreactor. PEHD = uncoated polyethylene high-density substrate. Conventional = simplified nonbiocidal FRC. AgNP and GO = nanocomposite FRCs with AgNPs and GO, respectively, at varying weight percentages (wt %). Data represent mean ± standard deviation from three independent replicates.

The application of the simplified nonbiocidal FRC led to a considerable reduction in biofouling, resulting in an average density of approximately 28 mm^–2^.

Nanocomposite coatings that incorporated AgNPs demonstrated a concentration-dependent decrease in the level of algal adhesion. The formulations containing 0.125 and 0.250 wt % AgNPs exhibited comparable fouling levels to the conventional coating, with mean cell densities of approximately 28 and 27 mm^–2^, respectively. In contrast, the 0.500 wt % AgNP coating achieved a significantly lower fouling level, reducing algal density to approximately 10 mm^–2^.

Coatings containing GO also displayed improved antifouling performance compared with both the uncoated control and the conventional formulation. The 0.125 and 0.250 wt % GO coatings yielded mean algal densities of approximately 21 and 19 mm^–2^, respectively, while the 0.500 wt % GO coating further decreased fouling to approximately 13 mm^–2^.

### Long-Term Field Immersion

The progression of fouling on PVC panels coated with three different types of coatings, simplified nonbiocidal FRC, nanocomposite FRC containing 0.500 wt % AgNPs, and nanocomposite FRC with 0.500 wt % GO, was observed during a continuous field immersion period of 7 months. The results are listed in Figure 5.

**Figure 5:**
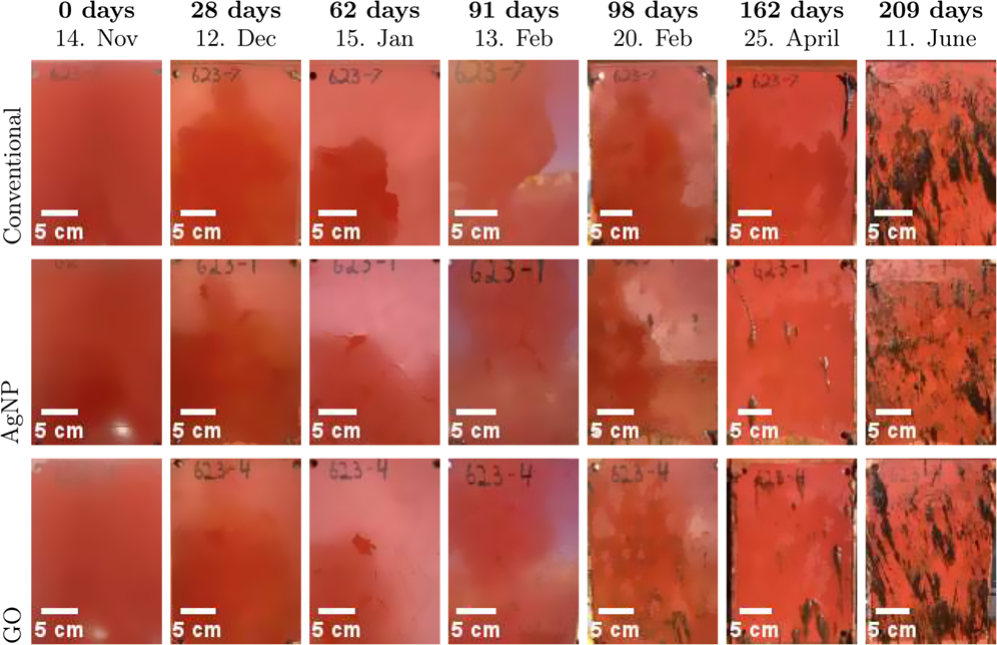
Representative photographs showing the time-dependent fouling accumulation over 7 months of continuous field immersion (14 November 2024 to 11 June 2025) for the simplified nonbiocidal FRC (conventional) (row 1), the nanocomposite FRC with 0.500 wt % AgNPs (row 2), and the nanocomposite FRC with 0.500 wt % GO (row 3).

Panels coated with simplified nonbiocidal FRCs (row 1) remained mostly free of visible fouling for the first 3–4 months of exposure. However, initial surface colonization became noticeable after 162 days, followed by a gradual increase in fouling coverage over time. By the 7 month mark, the surfaces showed significant fouling coverage from organisms, including biofilms and brown filamentous growth.

Panels coated with the nanocomposite FRC containing 0.500 wt % AgNPs (row 2) also exhibited minimal fouling accumulation during the first 3 months, though some fouling occurred slightly earlier than on the simplified nonbiocidal FRC. Some macrofouling became visible after 98 days, with coverage increasing progressively after this, ultimately resulting in extensive fouling similar to that observed on the simplified nonbiocidal FRC by the end of the observation period.

The panels coated with the nanocomposite FRC containing 0.500 wt % GO (row 3) also showed signs of fouling slightly earlier than the simplified nonbiocidal FRC, with visible biofilm development observed after just 98 days. The fouling coverage was greater than that of both the simplified nonbiocidal FRC and the AgNP-containing formulation. By month 7, the GO nanocomposite panels were characterized by nearly complete fouling coverage, as well.

Overall, the differences between the simplified nonbiocidal FRC and the nanocomposite coatings were minimal, as macrofouling occurred on all surfaces. Estimates of the average fouling coverage on the samples, along with the water temperature, are listed in Figure 6.

**Figure 6:**
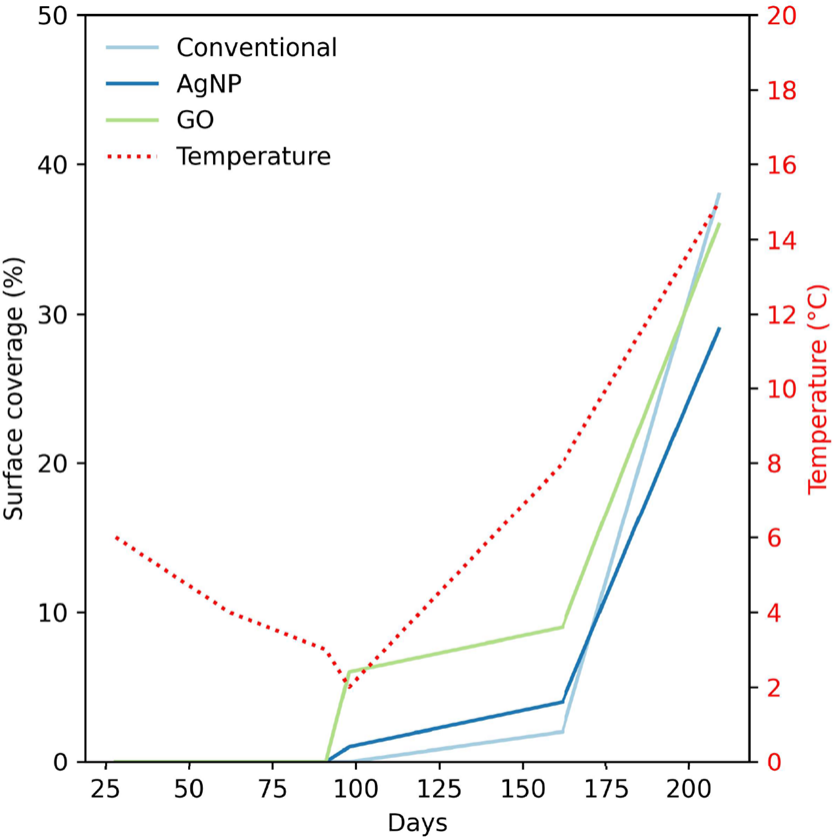
Estimated surface coverage on panels coated with the simplified nonbiocidal FRC and the nanocomposite FRCs with 0.500 wt % AgNPs or GO after 28, 62, 91, 98, 162, and 209 days of continuous field immersion from 14 November 2024 to 11 June 2025. The average temperature in water is shown as the dotted red line.

Rough estimates suggest that after 162 days of immersion, the simplified nonbiocidal FRC exhibited approximately 2% surface coverage, which increased to about 38% after 7 months. In contrast, the AgNP surface showed around 1% coverage after 98 days and approximately 4% after 162 days; by the end of 7 months, the surface coverage had risen to roughly 27%. For the GO surface, the coverage began at approximately 6% after 98 days and reached about 9% after 162 days. After 7 months, the surface coverage was estimated to be around 36%.

This notable increase in growth during the last few months of immersion aligns well with the seasonal transition from winter and spring to summer during this time. The average temperatures in the waters at Sandefjord in the winter months were 10, 6, and 4 °C for November, December, and January, respectively. Then, it stays at around 3 °C in February, with an increase to 8 and 15 °C for April and June, respectively. The estimated surface coverage on the coated panels follows the temperature trend, with little growth at low temperatures and then a gradual increase before rapidly increasing for the final month.

## Discussion

The nanocomposites in this study demonstrate a significant improvement in antifouling performance against microfouling in the lab compared to simplified nonbiocidal FRCs, most notably for higher filler concentrations. We see this trend of the increasing antifouling effect for increased filler content, likely due to the increase of exposed nanoparticles, even though the surface-exposed nanoparticles have the potential to disrupt the continuity of the FRC’s low-energy surface and introduce areas of higher polarity,^41^ negatively affecting the fouling release mechanism. However, the nanoparticles appear not to significantly change the surface energy since they are measured to be well within the statistical uncertainties, possibly due to the very low concentration used in these composites. The fouling release mechanism is expected to remain effective, as all surface energies fall comfortably within the necessary range of 20–30 mN/m. It follows that any increase in the antifouling efficacy must be a result of other factors; the enhanced antifouling efficacy observed under laboratory conditions is likely a result of a different antifouling mechanism, such as the biocidal effects of the nanoparticles. This is also supported by the results showing that fouling decreases with increased filler concentrations.

The cells appear to preferentially attach to areas where the nanoparticles are exposed to the surface. This behavior may result from the tendency of foulants to adhere to surface irregularities, which could be caused by the exposed nanoparticles. While there is limited research on the preferred settlement locations of cells on nanoparticle–polymer composite coatings, studies have explored the attachment of nanoparticles to bacterial surfaces. For instance, it has been shown that nanoparticles can adsorb onto algal cell walls, with positively charged particles exhibiting stronger adsorption due to electrostatic attraction to the negatively charged cell walls.^42,43^ Factors such as particle surface properties, medium conditions, and algal characteristics may influence this interaction. There is the potential to improve the antifouling properties of FRC nanocomposite coatings by increasing the nanoparticle load. This can enhance the biocidal effect, as the complementary fouling release effect and adequate hydrodynamic shearing helps to remove accumulated loosely attached dead foulants; increased amounts of biocidal particles on the surface may attract more cells to these specific locations on the surface and kill these cells, and the fouling release mechanism of the coating ensures that dead foulants are then removed. It is important to ensure an even distribution of nanoparticles and to prevent agglomeration, as this can reduce the efficiency of biocidal nanoparticles. Aggregation due to excessive nanoparticle loading in the polymer matrix corresponds to a greater adhesion strength of fouling organisms. It is crucial to balance the increase in nanoparticle load with proper dispersion within the coating to improve the biocidal effect of the nanocomposite coatings while maintaining their fouling release properties.

After 3–4 months of field immersion, the macrofouling observed on the surfaces is primarily a brownish algal filament, consistent with typical species such as *Pylaiella littoralis* and *Ectocarpus* spp. The panels were not investigated for microfouling; only macrofouling trends were considered during field immersion. Therefore, it is difficult to directly relate the microfouling performance in the lab to the macrofouling performance in real conditions, though some conclusions may be drawn on the biocidal effect of the nanocomposites by comparison to the simplified nonbiocidal FRC’s performance over time. Generally, the macrofouling was found to increase at the same rate as the temperature increases and along with the seasonal change. It is therefore likely that there is generally more fouling and not simply just the loss of the antifouling effect of the coatings. However, the fouling on the nanocomposite surfaces occurs at a slightly faster rate in the beginning than for the simplified nonbiocidal FRC. At the end of the 7—month period, macrofouling on the simplified nonbiocidal FRC had caught up to that on the GO composite, whereas the Ag composite exhibited less macrofouling than both. It appears that the composites attract more macrofouling at the early stage, as discussed previously; however, the biocidal effect of the coatings may enhance antifouling efficacy over time.

If the interfacial adhesion between the polymer and the nanoparticles is poor, these entrapped particles may be released from the coating surface and leach out to the environment.^44^ GO has shown relatively poor interfacial adhesion to PDMS, but this can be improved through functionalization or by introducing a nanoporous interfacial layer to enhance adhesion and compatibility.^45^ AgNPs are also subject to leaching from the polymer matrix over time when exposed to seawater, with the rate influenced by factors such as the size and shape of the particles, functionalization, and polymer type.^46^ The nanocomposites, which showed great initial microfouling prevention under laboratory conditions, may have lost this effect to some extent during long-term field exposure due to nanoparticle leaching or depletion. This could explain the earlier onset of the macrofouling. Poor interfacial adhesion may have compromised the integrity of the coating and resulted in the loss of the biocidal effect of the nanoparticle.^47^ This deterioration may also have led to decreased fouling release efficacy as the continuity of the low-energy surface is disrupted, introducing areas of higher polarity.^41^ Additionally, poor interfacial adhesion and compatibility could negatively impact the mechanical properties of the system, thereby compromising the operational potential of simplified nonbiocidal FRCs.

In terms of practical applications, the choice of substrates should be further considered. Though investigations into the antifouling performance of the coating surfaces show promise, design perspectives need to be taken into account. Limitations of the substrate, such as coating adhesion, should be carefully considered.

## Conclusions

Incorporating AgNPs and GO into silicone-based fouling release coatings can significantly enhance antifouling performance under controlled laboratory conditions with efficacy improving at higher nanoparticle loadings. The surface energies of the nanocomposite coatings remained within the optimal range for fouling release. Long-term field immersion performance of the nanocomposite coatings was comparable to that of the simplified nonbiocidal FRC, though the nanocomposites showed a slightly earlier onset of macrofouling. The AgNP-FRC composites showed slightly better antifouling efficacy in the field immersion compared to the GO-FRC and the simplified nonbiocidal FRC after 7 months. Substantial nanoparticle agglomeration and preferential algal attachment near exposed particles were found, reiterating the need for another fouling release mechanism in order to remove loosely attached dead cells. Though the addition of nanoparticles for boosting antifouling efficacy with both biocidal and fouling release strategies showed only marginal improvements, clear evidence of enhanced performance is seen. These findings highlight the importance of optimizing nanoparticle distribution and interface compatibility within the polymer matrix, such as through functionalization of the nanoparticles, to fully exploit both fouling release and biocidal antifouling mechanisms. Future work should focus on the connection between micro and macrofouling and the effect of the nanoparticles at each respective fouling stage, as well as dispersion strategies, nanoparticle functionalization, nanoparticle leaching, and long-duration field validation, to enable the development of robust, scalable, and environmentally sustainable antifouling coatings.

## Supplementary Material



## Notes

Additional results from the experimental work, such as the algae count from the samples exposed in the bioreactor, the measured contact angles and surface energies of all coated samples and the uncoated substrate, and the estimated surface roughness (PDF)
