# Supplementary material for: Laboratory and Field Evaluation of Graphene Oxide and Silver Nanoparticle-Enhanced Silicone Fouling Release and Biocidal Coatings for Marine Antifouling
Source: ACS Omega. 2026 Jan 21;11(4):5550–7. doi: 10.1021/acsomega.5c09101 (PMC12878346; doi:10.1021/acsomega.5c09101)
Supplement: Supplementary file 1 [file ao5c09101_si_001.pdf]

Supplementary Information

Laboratory and Field Evaluation of Graphene Oxide and Silver Nanoparticle-Enhanced Silicone Fouling Release and Biocidal Coatings for Marine Antifouling

Michael R. Kelly,*,† Olaug M. Aalen,†, Ingrid G. Hallsteinsen,† and Hilde L. Lein†

†*Department of Materials Science and Engineering, Norwegian University of Science and Technology NTNU, Sem Sælands vei 12, 7034 Trondheim, Norway*

E-mail: michael.r.kelly@ntnu.no

Table S1: Algae count for samples in bioreactor. Results were obtained by manually counting algae in images captured with the *Alicona Infinite Focus SL*.

Sample	Measurement	Number of algae [n]
	1	93
Uncoated PEHD	2	118
	3	91
	1	41
Simplified non-biocidal FRC	2	50
	3	47
	1	51
FRC-AgNP (0.125%)	2	43
	3	43
	1	43
FRC-AgNP (0.250%)	2	37
	3	40
	1	16
FRC-AgNP (0.500%)	2	17
	3	16
	1	41
FRC-GO (0.125%)	2	35
	3	30
	1	36
FRC-GO (0.250%)	2	29
	3	31
	1	27
FRC-GO (0.500%)	2	21
	3	18

Table S2: Measured contact angles for water and diiodomethane, together with calculated surface free energies, for all coated samples and the uncoated control substrate.

Sample	Contact angle [°]		Surface free energy [mN/m]
	Water	Diiodomethane	
Uncoated PEHD	105.8 ± 0.2	59.3 ± 2.0	29.0 ± 0.9
Simplified non-biocidal FRC	111.5 ± 0.1	62.2 ± 0.8	27.7 ± 0.1
FRC-AgNP (0.125 wt%)	111.0 ± 0.6	62.2 ± 0.5	27.3 ± 0.3
FRC-AgNP (0.250 wt%)	110.2 ± 0.5	60.0 ± 0.3	28.6 ± 0.2
FRC-AgNP (0.500 wt%)	107.7 ± 0.5	60.2 ± 0.1	28.5 ± 0.1
FRC-GO (0.125 wt%)	109.9 ± 0.2	61.5 ± 1.2	27.7 ± 0.7
FRC-GO (0.250 wt%)	111.3 ± 0.4	60.9 ± 0.3	27.9 ± 0.2
FRC-GO (0.500 wt%)	110.9 ± 0.6	59.6 ± 0.9	28.8 ± 0.5

Table S3: Estimated surface roughness for all coated samples.

Sample	Average Roughness [μm]
Simplified non-biocidal FRC	0.89
FRC-AgNP (0.125 wt%)	0.47
FRC-AgNP (0.250 wt%)	0.42
FRC-AgNP (0.500 wt%)	0.72
FRC-GO (0.125 wt%)	0.49
FRC-GO (0.250 wt%)	0.54
FRC-GO (0.500 wt%)	0.49

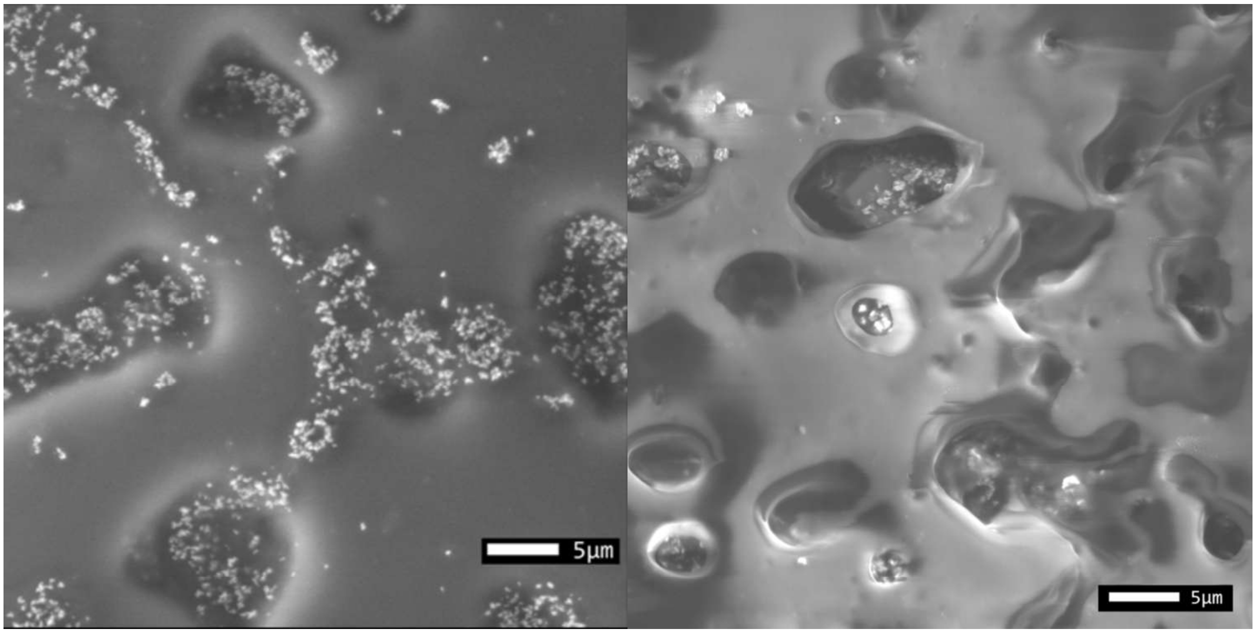

(a) FRC-GO (0.250 wt%) (b) FRC-AgNP (0.500 wt%)

Figure S1: A selection of coating surfaces after spray deposition.
